# Impact of interactive multi-media learning for physicians in musculoskeletal education – a pilot study

**DOI:** 10.1186/s12909-022-03746-4

**Published:** 2022-10-12

**Authors:** Veronica Wadey, Tosan Okoro, Thrmiga Sathiyamoorthy, David Snowdon, Heather McDonald-Blumer, Alfred Cividino, Deborah Kopansky-Giles, David Levy, Risa Freeman, Jodi Herold, Douglas Archibald

**Affiliations:** 1 Division of Orthopaedic Surgery, University of Toronto, Toronto, Canada; 2 Department of Arthroplasty, Robert Jones and Agnes Hunt Orthopaedic Hospital, Oswestry, UK; 3 Temerty Faculty of Medicine, University of Toronto, 1 King’s College Circle, Toronto, ON M5S 1A8 Canada; 4 Applied Clinical Pharmacology, University of Toronto, Toronto, Canada; 5 Division of Rheumatology, University of Toronto, Toronto, Canada; 6 McMaster University, Hamilton, Canada; 7 Department of Research, Canadian Memorial Chiropractic College, North York, Canada; 8 Department of Family Medicine, McMaster University, Hamilton, Canada; 9 Department of Family & Community Medicine, University of Toronto, Toronto, Canada; 10 Faculty of Medicine, University of Ottawa, Ottawa, Canada

**Keywords:** Musculoskeletal health, Multi-media, Interactive learning

## Abstract

**Background:**

The aim of this educational study was to investigate the use of interactive case-based modules relating to the screening and identification of early-stage inflammatory arthritis in both online technology (OLT) and paper (PF) formats with identical content.

**Methods:**

Forty learners from family medicine or rheumatology residency programs were recruited. Content pertaining to a “Sore Hands, Sore Feet” (SHSF) and Gait Arms Legs Spine (GALS) screening tool modules were selected, reviewed and developed based on a validated curriculum from the World Health Organization and Canadian Curriculum for MSK conditions. Both the SHSF module and GALS screening tool were assessed via a randomized control trial. Assessments were completed during an orientation with all learners; then prior to the intervention (T1); at the end of the module (T2) and 3 months following the modules (T3) to assess retention. Focus groups were conducted to determine learners’ satisfaction with the different learning formats. Baseline data was collated, and analysis performed after randomization into the PF (control) and OLT (experimental) groups. Repeated measures ANOVA was used for statistical analyses.

**Results:**

Forty participants were recruited and randomized into the PF or OLT group (*n* = 20 each). At 3 months, there were *n* = 31 participants for SHSF (PF *n* = 19, OLT *n* = 12) and *n* = 32 for GALS (PF *n* = 19, OLT *n* = 13). There was no significant difference between the OLT and PF groups in both analyses. A significant increase in scores from Pre- to Post-Module in SHSF (F (1, 18) = 24.62. *p* < .0001) and GALS (F (1, 30) = 40.08, *p* < .0001) were identified to suggest learning occurred with both formats. The repeated measures ANOVA to assess retention revealed a significant decrease in scores from Post-Module to Follow-up for both learning format groups for SHSF (F (1, 29) = 4.68. *p* = .039), and GALS (F (1, 30) = 18.27. *p* < .0001) suggesting 3 months may be too long to retain this educational information.

**Conclusions:**

Both formats led to residents’ ability to screen, identify and initially manage inflammatory arthritis. The hypothesis is rejected because both OLT and PF groups demonstrated significant learning during the process regardless of format. It is important to emphasize that from T1 (pre-module) to T2 (post-module), the residents demonstrated learning regardless of group to which they were assigned. However, learning retention declined from T2 (post-module) to T3 (three-month follow-up). Regular review of knowledge may be required earlier than 3 months to retain information learned. This study may impact educational strategies in MSK health.

**Trial registration:**

This study did not involve “patients” rather learners and as such it was not registered.

## Introduction

Historically, Inflammatory arthritis has been under diagnosed and initial management often delayed. A focus should be on educating health care providers to be able to screen for arthritis; identify and initially manage patients with inflammatory arthritis [1]. Patients affected with inflammatory arthritis need timely and aggressive care in order to optimize the benefits currently available to them, and the assessment skills of our frontline health care providers are critical. Millions of healthcare dollars may be saved if frontline healthcare providers are able to correctly identify and refer patients with inflammatory arthritis early. Additionally, if this online learning methodology can effectively educate health professionals to provide early detection and treatment of inflammatory arthritis, it may be adapted to other critical healthcare issues.

The primary rationale for this focus is the high current and projected burden of illness related to musculoskeletal (MSK) conditions [2–4]. MSK conditions affect approximately 1.71 billion people worldwide and are the leading contributor to severe chronic pain and physical disability [5]. They are the second most common reason for the need to see a physician [6], and the most common cause of health related problems leading to a person’s inability to work [5]. The economic burden of MSK conditions is enormous, both directly through health expenses and indirectly through loss of productive years [7].

The impact of arthritis is significant in Canada. The Alliance for the Canadian Arthritis program (ACAP) stated in their summary notes from the National Summit on Standards for Arthritis Prevention and Care in the fall of 2005 that “the area of arthritis, which impacts more than 4 million Canadians of all ages and is the country’s leading cause of deformity and long-term disability, is in urgent need of major reform, or the situation will get worse.” Since the summit, the situation has worsened, with the 2018 report on the Status of Arthritis in Canada citing that over 6 million Canadians have arthritis [8]. The report also stated that the prevalence of arthritis is increasing, and that by 2040 over 9 million Canadians will have arthritis [8]. The burden of arthritis is on the rise, and identifying those with inflammatory arthritis early so that appropriate treatment may be initiated to protect joints and increase mobility is critical. The key to increasing awareness, prevention, and access to care for individuals with arthritis involves the education and training of health care providers. This topic was identified as a priority item at the 2005 National Summit [9]. Primary care physicians’ investigation of inflammatory arthritis was in accordance to current standards, however the rates of referral to rheumatologists and other health care professionals is reported to be low, especially in cases presenting early [10]. In addition, rheumatologists appear to face significant barriers to providing adequate care to patients [11]. Postgraduate training may be the most important and modifiable variable to improve physician management of MSK conditions [12], and in particular inflammatory arthritis.

The Bone and Joint Decade (BJD); declared by the World Health Organization on January 13^th^, 2000 had a target to improve the quality of life for people with MSK disorders worldwide, aiming to increase education and training of health care providers at all levels [13]. MSK curricula are therefore required in various residency training programs managing patients with these conditions. The problem is the lack of curricula with a specific focus on musculoskeletal (MSK) content [14–22]. The aim of this study was to determine if working through musculoskeletal educational content delivered through on-line (OLT) multi-media tools and paper format would improve participants’ knowledge, skills and satisfaction in learning how to identify patients with early-stage inflammatory arthritis when compared to learners using paper format (PF). We are striving to refine not only our educational tool development for content and interactivity but also to be innovative in our methods to support distributive learning techniques.

## Methods

The study design consisted of a randomized control trial of educational research using a single blinded randomization technique comparing learners using on line technology (OLT—multi-media) and paper format (PF) as methods for conveying identical educational information. The participants consisted of 40 resident learners from either family medicine or rheumatology residency training programs both considered to be possible frontline healthcare providers of MSK conditions in a large institution within North America. These programs were specifically identified for this study as family medicine residents are frontline physicians in a particular training program that requires them to successfully identify individuals and refer to subspecialty care individuals with inflammatory arthritis. Rheumatology residents represent residents who are required to identify, diagnose and manage individuals with inflammatory arthritis. This is a pilot study for the purpose of establishing a proof of concept of an interactive learning and assessment tool therefore a power calculation was not completed. Ethics to complete this research was received from the University.

The following information outlines the evolution on how the first module for the MSK curriculum evolved and in particular, outlines the process by which the module on “Sore Hands, Sore Feet” (SHSF) and Gait Arms Legs Spine (GALS) screening tool were developed and assessed.

### Bone and joint decade undergraduate curriculum group (BJDUCG) need’s assessment

The BJDUCG completed a needs assessment of core curriculum recommendations on MSK conditions [23], with a focus on medical school education [23]. Medical experts representing 29 nations developed these recommendations. This was completed as a World Health Organization education initiative.

### National needs assessment

Following the BJDUCG’s medical school curriculum recommendations, a national needs assessment at the postgraduate level was then conducted to determine if the same MSK curriculum applied to the postgraduate level of training among numerous potential frontline health care disciplines within Canada. This initiative was funded by the Royal College of Physicians’ and Surgeons of Canada Medical Education Travelling Fellowship [1]. The national needs assessment involved 165 educational thought leaders representing 77 academic postgraduate training programs, 6 disciplines in medicine at 16 accredited universities across Canada [1] for the purpose of determining what topics should be highlighted for a proposed MSK health website. The opinion was to consider innovative ways of educating and assessing health care providers using multimedia technologies in the form of on-line learning. The development of these technologies would be to support an MSK health website targeted to address the educational needs of health care providers [24].

### National consensus workshop

A national consensus workshop followed the nation-wide needs assessment for a 1.5-day inaugural meeting consisting of participants representing multiple disciplines across Canada, and was completed in 2007. Participants of this meeting included representatives from the: RCPSC Specialty Committee of Rheumatology, Canadian College of Family Physicians, Canadian Chiropractic College, and Canadian Academy of Health Sciences, as well as rehabilitation experts, statisticians, instructional designers, technology and evaluation template experts, and patient representatives. These individuals were divided into the medical expert content team and the design support team. The content team consisted of patient representatives and content experts in the areas of screening for Arthritis, simulation, and medical experts. The design team consisted of statistical leads, evaluation templates experts, systems architects, and instructional designers. Our goal was to assemble and evaluate the complimentary skill sets of the major stakeholder groups in a cost-effective manner [25].

The consensus workshop verified that content in the form of case-based simulations pertaining to the approach to symmetrical joint pain and stiffness should be developed as the first case based module when considering all topics among the MSK curriculum. A website was constructed and made universally accessible as a learning and assessment tool for any health care provider locally, nationally, and possibly beyond. The main deliverable after the national consensus meeting was a working template of content to be assembled into a case based scenario, the outcome measures to be implemented into the education and assessment components of the module and the design format for the online learning process. Upon completion of the meeting, a small working group was assembled to review the summary of the proceedings and to formulate the case simulation based on a “real case presentation”. The chair of the RCPSC Specialty Committee of Rheumatology provided this case. The scenario pertained to a young woman experiencing sore hands and sore feet. The tool was to be developed based on the evidence and designed to educate and assess the participants’ ability to approach a patient with symmetrical joint pain and stiffness. This module would incorporate interactive education and assessment exercises that would function to augment, not replace, clinical encounters [26].

The group consensus was to develop a module to be a “proof of concept” for a pilot study. Principles used to assemble the content included: 1) being evidence based; 2) potentially connecting to existing knowledge networks; and 3) designed specifically by an inter-professional team of health care experts and patients (consumers).

Every condition in MSK health begins with a clinical presentation of a particular problem. The content pertaining to this module was formatted in a similar manner to a “prediction model” on how to detect arthritis early [27], and was developed based on the “key features” and critical decision making [28]. Content included educating participants on: 1) how to screen for arthritis using a previously validated gait, arms, legs, spine (GALS) screening examination; 2) the hallmarks of history and physical examination of joints and axial skeleton in patients presenting with symmetrical joint pain and stiffness; 3) constructing a differential diagnosis and working diagnosis based on the history and physical examination; 4) radiographs and laboratory investigations required to formulate a working diagnosis; 5) how to interpret the results of these investigations and the synthesis of the data obtained; 6) formulating an initial management plan; and 7) how to appropriately refer to subspecialty care.

### Multi-disciplinary focus group

A focus group of content experts met on 15 occasions to assemble the content and create the learning and assessment tools for the module pertaining to inflammatory arthritis and more specifically, “Sore Hands, Sore Feet”. Video productions of the patient presenting with sore hands and feet focusing on communication skills and physical examination were also completed to augment the content template. The storyboard with the e-learning developers for both interactive educational and assessment tools was developed with specific interactive learning modules for the Gait, Arms, Legs, Spine (GALS) screening for arthritis which was designed to be embedded into the Sore Hands, Sore Feet (SHSF) module. These learning experiences were meant to improve health care providers’ ability to screen for arthritis; to assess, diagnose and initially manage an individual with inflammatory arthritis and; to learn how to refer to subspecialty care. These education modules were developed with a team of health care providers representing physicians, surgeons, residents, and other health care providers such as physiotherapists and chiropractors as well as a patient representative. We complemented the medical team with a group of educational researchers and technology specialists. Paper format of the GALS and SHSF were then developed to reflect a similar learning experience to that of the on-line format with respect to content provided and was accompanied by the same pre- and post- module learning assessments.

### Content review

A full content review was performed with eighty Canadian health care providers representing medical students, physicians, surgeons, physical therapists, occupational therapists, nurses, and chiropractors to review online content for accuracy and the tool for functionality. Pre/post-test measures for GALS and SHSF were completed for the following areas: 1) Response patterns for items pre- and post- tests; 2) difficulty indices for items pre- and post- tests; 3) Item-total correlation coefficients pre- and post- tests and; 4) Comparison of mean scores pre- and post- module.

### Randomized control trial (Pilot Study)

The information from this content review generated the main outcome measures used for the randomized control trial (RCT) pilot study. An RCT pilot study was the preferred method of assessment to reduce the influence of preference for learning style. The inclusion criteria were volunteer residents from a family medicine training program and a rheumatology training program specifically selected as potential health care providers most likely to see patients with this medical condition. There were no exclusion criteria. For each of the educational experiences (GALS screening tool that was incorporated into the SHSF interactive learning module), the group of 40 residents was randomly divided into either the paper format (PF) control group (*n* = 20) or the online technology (OLT) experimental group (*n* = 20).

**Fig. 1:**
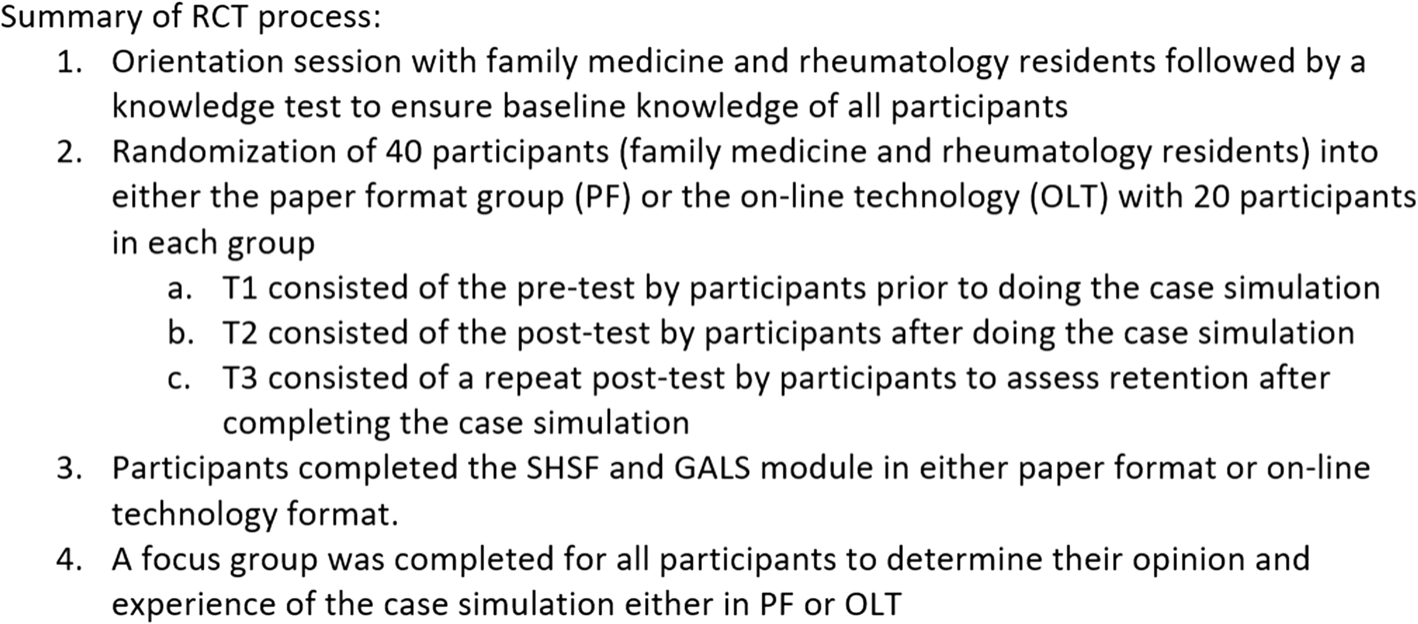
Design of a learning module

The GALS screening tool was incorporated into the overall SHSF module and was also assessed independently for its usefulness as a screening tool for arthritis. Participants experienced an orientation session with pre/post-test questionnaires and analyses were completed (Fig. 1).

**Fig. 2:**
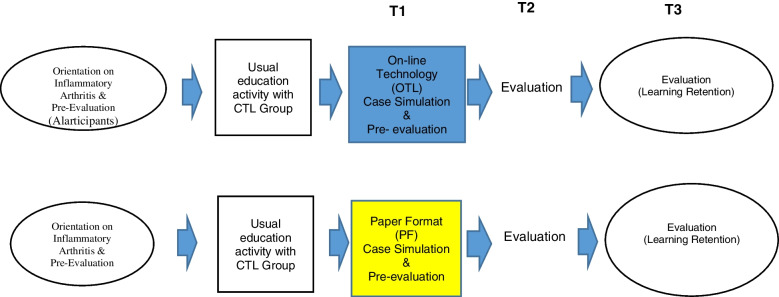
Schematic of the evaluation process for participants in experiential and control groups

Thirty-two subjects completed the GALS assessment. Repeated Measures ANOVA was used to assess the extent to which there was an effect of format on learning from Time 1 (Pre-Module) to Time 2 (Post-Module). A separate Repeated Measures ANOVA was run to determine the effect of format on retention from Time 2 to Time 3 (3 month Follow-up). Focus groups were completed (Fig. 2).

Similarly, participants were randomized via a single blinded randomization technique into the SHSF module to either OLT or PF groups. All participants experienced an orientation session with pre/post-test questionnaires and analyses were completed. Thirty-one subjects completed the SHSF learning module. Data was analyzed based on: Time 1 (Pre-Module), Time 2 (Post-Module) and Time 3 (3 month Follow-up). Repeated Measures ANOVA was used to assess the extent to which there was learning in the PF group from Time 1 to Time 2. One-way ANOVA was used to assess the effect of learning format on Post-Module test scores. A separate Repeated Measures ANOVA was run to determine the effect of format (OLT versus PF) on retention from Time 2 to Time 3.

**Fig. 3:**
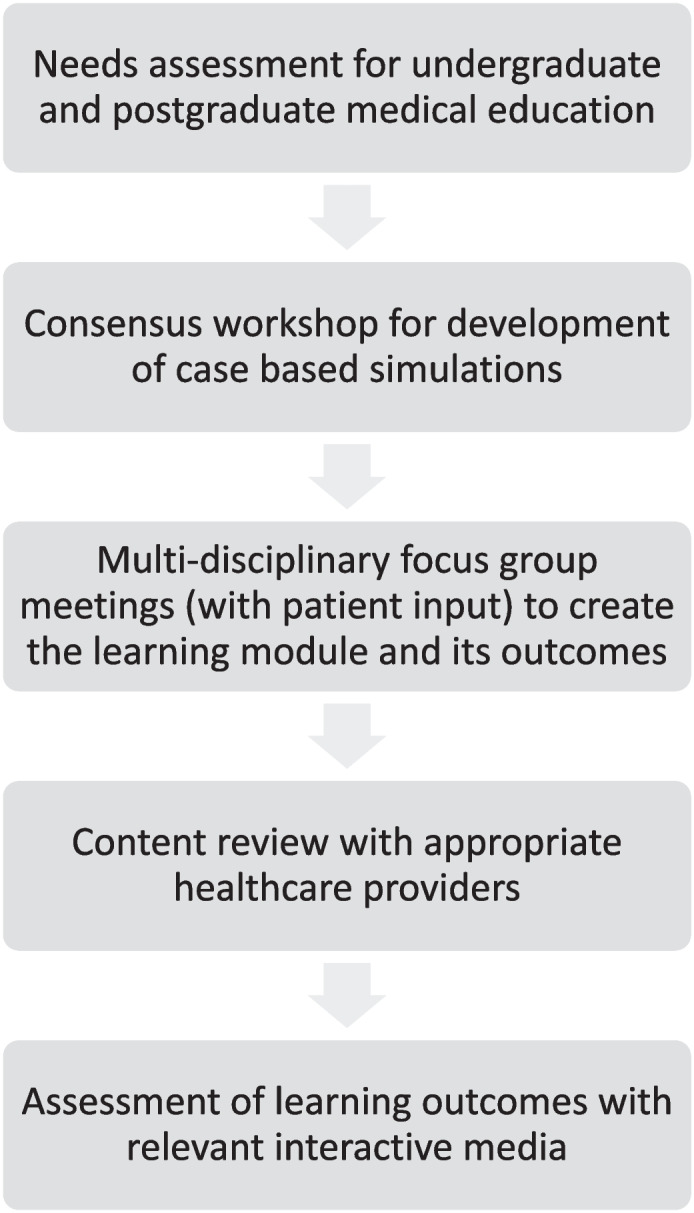
Consort 2010 flow diagram

A summary of the template used to develop the interactive educational learning models for use either by on line or paper format was created. The template may be generalized to other MSK conditions (Fig. 3).

## Results

### Participants

**Fig. 4:**
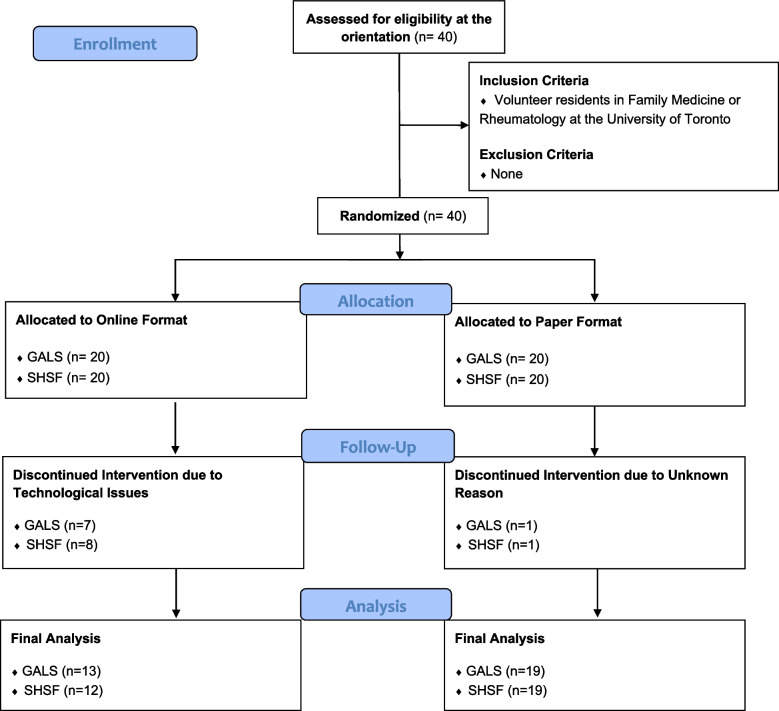
Consort 2010 flow diagram

A total of 40 participants were recruited to this study. The 40 participants were randomized by single blinded technique into either the OLT group or the PF group for each of the educational modules GALS screening tool and the overall SHSF module pertaining to inflammatory arthritis. Eight participants were lost to follow-up in GALS, and 9 participants were lost to follow-up in SHSF. Therefore, 32 subjects participated in the GALS portion of the module and 31 participants participated in the SHSF portion of the module. Refer to Fig. 4, which displays the RCT flow diagram adapted from CONSORT.

### Demographics

An analysis was completed after the initial education session provided to all participants to ensure baseline knowledge was at least similar at the beginning of the study. There appeared to be no baseline differences deemed to be significant among the family medicine residents or rheumatology residents. An orientation session on inflammatory arthritis with pre/post-tests demonstrated no significant difference in post orientation test scores among the residents assigned to the online (OLT)group and those assigned to the paper formats (PF) group (means of 2.60 points out of 4 for the OLT group; 2.76 points out of 4 for the PF). Thus all residents were treated equally from the outset of this pilot study.

### Gait arms legs spine (GALS) screening for arthritis

There were 40 participants randomized in GALS module. Twenty were randomized to the PF group and 20 to the SHSF group.

In the OLT group 7 of the 20 participants were lost to follow-up. Data was analyzed based on 13 participants in the OLT experimental group. In the PF group 1 of the 20 participants was lost to follow-up. As such data was analyzed based on 19 in the PF control group. Table 1,2,3 shows the mean scores at pre-module, post-module, and follow-up for the GALS module, and demonstrates that both groups learned from the interactive educational experience offered by completing the module regardless of the method of learning, and that there was no significant difference between the OLT and PF group. It can also be seen in Table 1,2,3 that both OLT and PF groups experienced a decline in test scores at the 3 month follow-up, however there was no significant difference in the extent to which each group did so.

**Table 1:** Three assessment time points mean scores, for both online and paper-based formats of the GALS module

Time	Format	Mean (/16)	Std. Deviation	N (number of subjects)
**Pre-Module**	Online	2.23	1.092	13
	Paper	2.16	1.214	19
	Total	2.19	1.148	32
**Post-Module**	Online	3.77	1.013	13
	Paper	3.16	1.119	19
	Total	3.41	1.103	32
**Follow-up**	Online	3.15	1.345	13
	Paper	2.16	1.344	19
	Total	2.56	1.413	32

**Table 2:** Test within subjects – GALS (T1 – T2)

Source	Time_1v2_GALS	Type III Sum of Squares	Degrees of Freedom	Mean Square	F	Significance
Time	Linear	24.869	1	24.869	40.078	.000
Time* Format	Linear	1.119	1	1.119	1.803	.189
Error(Time GALS)	Linear	18.615	30	.621

**Table 3:** Test within subjects – GALS (T2 – T3)

Source	Time_2v3_ GALS	Type III Sum of Squares	Degrees of Freedom	Mean Square	F	Significance
Time	Linear	10.071	1	10.071	18.268	.000
Time* Format	Linear	.571	1	.571	1.036	.317
Error (TimeGALS)	Linear	16.538	30	.551		

The GALS screening tool demonstrated significant increases in mean scores from time 1 (45% for the OLT group; 43% for the PF group) to time 2 (75% for the OLT group; 63% for the PF group), demonstrating that both groups learned from the module.

The first Repeated Measures ANOVA (to assess learning) revealed that there was a significant increase in scores from the pre-module to the post-module for both online and paper format groups, F (1, 30) = 40.08, *p* < 0.0001. However, there was not an effect of format on the increase in scores. The second Repeated Measures ANOVA (to assess retention) revealed that there was a significant decrease in scores from post-module to follow-up for both format groups. F (1, 30) = 18.27. *p* < 0.0001. There was not a statistically significant effect of format on scores over time. (Tables 1, 2, 3).

### Sore hands, sore feet (SHSF) learning module

Forty participants were randomized in the SHSF educational learning module. Twenty participants were randomized to the OLT group and 20 participants to the PF group.

Eight participants were lost to follow-up in the OLT group because we did not have a complete data set for this study group. These eight participants did not complete the pre-module questionnaire because of a technological prompting failure ensure all participants completed the pre-test at the beginning of the module. This resulted in missing data. As such, data was analyzed based on 12 participants in the OLT experimental group. Similarly, 1 participant was lost to follow-up in the PF group. As such, data was analyzed based on 19 participants in the PF control group. All participants completing both the PF and OLT interactive learning modules completed the initial base-line assessments of knowledge pertaining to inflammatory arthritis and the post-module assessments. All 19 participants in the PF group completed the pre-module assessment. The 12 participants in the OLT group did not complete the pre-module assessment.

The overall mean baseline scores at the time of the initial orientation where education on inflammatory arthritis was provided to all participants demonstrated 65% for the OLT group and 69% for the PF group. The mean post-module scores was 77% for the OLT group and 80% for the PF group. This data suggests that a comparable amount of interactive learning was experienced by participants in both OLT and PF groups.

The Repeated Measures ANOVA to assess learning revealed that there was a significant increase in the scores from Pre-Module to Post-Module for the Paper-format (PF) learning group with *p* < 0.0001 (Tables 4,5,6). The One-way ANOVA showed that there was no effect of learning format on Post-Module test scores (Table 4, 5, 6).

**Table 4:** Pre-module, post-module and follow-up SHSF test scores, online and paper-based learning formats

Time	Format	Mean (/16)	Standard. Deviation	N (number of subjects)
**Pre-Module**	Paper	11.21	2.30	19
**Post-Module**	Online	12.25	0.75	12
	Paper	12.79	2.20	19
	Total	12.58	1.78	31
**Follow-up (Retention)**	Online	11.67	1.78	12
	Paper	12.16	2.29	19
	Total	11.97	2.09	31

**Table 5:** Post-module tests of within subjects

Source	Time	Type III Sum of Squares	Degrees of Freedom	Mean Square	F	Significance
time	Linear	5.428	1	5.428	4.675	.039
time * Format	Linear	.009	1	.009	.007	.932
Error(time)	Linear	33.669	29	1.161

**Table 6:** Post-module tests of between-subjects effects

Source	Type III Sum of Squares	Degrees of Freedom	Mean Square	F	Significance
Intercept	8780.552	1	8780.552	1347.765	.000
Format	3.907	1	3.907	.600	.445
Error	188.932	29	6.515		

The Repeated Measures ANOVA to assess retention revealed that there was a small but statistically significant decrease in scores from Post-Module to follow-up for both learning format groups with *p* = 0.039, but there was not a statistically significant effect of format on the decrease in scores over time. Table 4,5,6 shows the decrease in mean scores and standard deviations post-module to follow-up for both the OLT and the PF groups.

### Focus group results

The participants indicated through a focus group that they were better equipped to identify and initially manage inflammatory arthritis, regardless of the interactive learning method experienced. The interactive process of learning was perceived by the learners to be effective and did replicate a “real” experience for the learner. The learners perceived that the case flow was realistic; synthesized patient care information that was pertinent to their practice; optimized their learning in a very reasonable time frame and; provided them with a learning activity that they would do again if provided the opportunity.

## Discussion

This study investigated the use of 2 online interactive learning modules (GALS and SHSF) in educating health professionals to screen, diagnose, and initially manage a patient with inflammatory joint disease, and to appropriately refer patients to subspecialty care. It also compared this approach with a paper format containing the same information to determine the effect that this had on participants. The hypothesis was that the online case simulation would be superior to the paper format for both modules and that all participants would feel more comfortable addressing early-stage inflammatory arthritis after completion of the modules.

The case simulation module pertaining to the detection, initial management and referral to subspecialty care of a patient with inflammatory arthritis (SHSF) demonstrated a significant increase in scores from Time 1 to Time 2 for the PF group. This may suggest that more traditional interactive learning methods using paper format are an effective learning tool. This may be due to: 1) learner preference and; 2) the advantage that spatial landmarks provide [29]. Research suggests that there is a link between spatial representation of a text and reading comprehension. Readers who can remember the order of information relative to fixed spatial cues generally score higher on comprehension tests [30]. Cues such as recalling that the description of a certain technique was located at the top of a page may help the reader mentally reconstruct the information and improve memory and the ability to recall specific pieces of information [31].

Participants completing the OLT interactive learning experience, did not complete the pre-module assessment due to a probable technological programing issue. Participants were required to complete the pre-assessment, however, all participants failed to navigate themselves to actually enter the link to complete the pre-module assessment. Thus, this data was not collected in the OLT group. As such, there is no data showing an improvement from pre-module to post-module. However, there is data showing improvement in learning after completing the learning module in the OLT group. In addition, the 4-item pre-test that was completed by all participants at the time of the initial orientation session with all participants indicated that there was no significant difference in the baseline level of knowledge as assessed by that pre-test between the OLT group and the PF group. The similarity of the mean baseline scores (65% for the OLT group; 69% for the PF group), in conjunction with the similarity of the mean post-module scores (77% for the OLT group; 80% for the PF group), makes it reasonable to assume that there was a comparable amount of learning done by the 2 groups. In addition, the mean pre-module score for the PF group was 70% (compared to a baseline score of 69%), which further strengthens the assumption that the baseline assessment was an appropriate measure of the participants’ knowledge prior to exposure to the module which by design was the intervention used to enhance knowledge in both the OLT and PF groups.

The module focused on screening for arthritis using the GALS screening tool demonstrated significant increases in scores from time 1 (45% for the OLT group; 43% for the PF group) to time 2 (75% for the OLT group; 63% for the PF group), demonstrating that both groups learned from the module. These results are of importance because there is evidence to show that the GALS screening tool is effective in detecting arthritis when used properly [32, 33]. One study in particular taught primary care physicians to perform the GALS screening test by watching an instructional video, then subsequently had them assess 99 patients, and compared their results with rheumatologists who examined the same patients [32]. There was found to be a significant level of agreement between them, particularly when assessing gait and arms. There is also research to show that other frontline healthcare providers (e.g. physiotherapists, nurse practitioners) can appropriately incorporate the GALS screening exam into their practices [33, 34] therefore increasing the likelihood that a patient with inflammatory arthritis will be identified earlier.

The significance of this study goes beyond the comparison of the interactive learning method used. The aim of the BJD was to improve the quality of life for people living with MSK disorders. A lack of curriculum with a specific focus on MSK content was identified, and the methodology outlined earlier was created in part to address this issue. The Sore Hands, Sore Feet (SHSF) module was assembled with a focus on case-based simulations, to be assessed using two interactive methods of learning (PF and OLT). Thus, a subsequent RCT pilot study was completed to examine if an interactive educational experience pertaining to inflammatory arthritis (SHSF module and the GALS module) on the education of healthcare workers in the identification, initial management and referral to subspecialty care of patients with inflammatory arthritis was completed.

The strengths of this study included: 1) A single blinded randomized educational study was completed; 2) It was a randomized control trial pilot study assessing learning methods and the impact on improving knowledge and skills for health care providers managing an MSK condition; 3) It demonstrated that interactive learning experiences associated with “authentic” clinical experiences may be an optimal learning strategy; 4) Learners indicated that both paper format and on-line learning platforms may be useful in optimizing learning as long as case simulation is within the theme of module development and; 5) A hybrid model for education may reflect future medical education environments.

The limitations of this study included: 1) Unintended consequences of technology were experienced and the need to be aware that constant adaptations to advances in technology will be required for future studies and; 2) Participants were less likely to be lost to follow-up with the PF format than the OLT. This may suggest a more familiar method of learning at the time of this study was with paper format. However with education and increased exposure, technology and the convenience of having access to technology may result in more optimal learning experiences.

The increasing burden on the healthcare system due to musculoskeletal disorders may be lessened with early identification and referral to subspecialty care of any patients with inflammatory arthritis. Innovative education methods designed to improving postgraduate training by the development and integration of specific case-based modules may be a significant step to enhance educational offerings in postgraduate medical education.

## Conclusions

This study indicated that interactive case-based learning using either paper format or an on-line multi-media platform increases knowledge and skills pertaining to learners’ abilities to: 1) identifying inflammatory arthritis; 2) initially manage the condition and; 3) appropriately refer to subspecialty care. In addition, a template to further inform the successful development, assessment and implementation of other MSK educational and assessment tools resulted from this work. This may potentially impact how we strive to enhance education of our frontline health care providers locally, nationally and globally.

## Data Availability

The raw data that supports the findings of this study are no longer available. However, the full summary of the overall analysis is available and has been submitted as a related file.
